# Comparative cephalometric study of the airways between different ethnic groups with normal occlusion

**DOI:** 10.1590/2177-6709.29.2.e2423206.oar

**Published:** 2024-05-20

**Authors:** Genesis ZAMBRANO, Jose Gregorio Pelayo GUERRA, Laura Dias SOVIERO, Renan Morais PELOSO, Felicia MIRANDA, Karina Maria Salvatore de FREITAS, Arnaldo PINZAN, Marcos Roberto de FREITAS

**Affiliations:** 1 Instituto Mondelli de Odontologia, Ortodontia (Bauru/SP, Brazil).; 2 Universidade de São Paulo, Faculdade de Odontologia de Bauru, Departamento de Ortodontia (Bauru/SP, Brazil).; 3 Universidade Ingá, Departamento de Ortodontia (Maringá/PR, Brazil).

**Keywords:** Ethnicity, Cephalometry, Nasopharynx, Oropharynx, Etnia, Cefalometria, Nasofaringe, Orofaringe

## Abstract

**Objective:**

This study aimed to compare the nasopharynx and oropharynx airway dimensions of Caucasians, Blacks, Japanese, Japanese Brazilians, and Black Caucasians.

**Methods:**

A sample of 216 lateral radiographs of untreated young Brazilian subjects (mean age of 12.94 years; SD 0.88) were divided into five groups: Black Caucasian, Black, Caucasian, Japanese, and Japanese Brazilian. Lateral radiographs were used to measure the oropharynx (from the midpoint on the soft palate to the closest point on the anterior pharyngeal wall) and the nasopharynx (from the intersection of the posterior border of the tongue and the inferior border of the mandible to the closest point on the posterior pharyngeal wall). Analyses of variance (ANOVA) and Tukey’s test were performed (*p*< 0.05).

**Results:**

The linear dimension of the oropharynx was similar among the different ethnic groups. Caucasian individuals presented a significantly greater linear dimension of the nasopharynx than Black Caucasian and Black individuals.

**Conclusions:**

All the groups had similar buccopharyngeal values. However, Caucasian individuals had significantly higher values when compared to Black Caucasians and Black individuals.

## RESUMO

**Objetivo:**

Este estudo teve como objetivo comparar as dimensões das vias aéreas da nasofaringe e da bucofaringe de leucodermas, melanodermas, xantodermas, feodermas e nipo-brasileiros.

**Material e métodos:**

Uma amostra de 216 telerradiografias laterais de jovens brasileiros não tratados (idade média de 12,94 anos; DP 0,88) foi dividida em cinco grupos: melanodermas, leucodermas, xantodermas, feodermas e nipo-brasileiros. As telerradiografias foram utilizadas para medir a bucofaringe (do ponto médio do palato mole até o ponto mais próximo da parede anterior da faringe) e a nasofaringe (da intersecção da borda posterior da língua e da borda inferior da mandíbula até o ponto mais próximo). Foram realizadas análises de variância (ANOVA) e teste de Tukey (*p* < 0,05).

**Resultados:**

A dimensão linear da bucofaringe foi semelhante entre os diferentes grupos étnicos. Indivíduos leucodermas apresentaram dimensão linear da nasofaringe significativamente maior do que indivíduos feodermas e melanodermas.

**Conclusões:**

Todos os grupos apresentaram valores da bucofaringe semelhantes. No entanto, os indivíduos leucodermas apresentaram valores significantemente maiores, quando comparados aos feodermas e melanodermas.

## INTRODUCTION

Studies comparing different ethnic groups have been reported since the 18^th^ century in Egypt, where the population was divided by skin color into red, yellow, black, and white.^1^ Considering the structural, craniofacial, and dental differences between ethnic groups, it is not recommended that normative patterns of cephalometric variables be inadvertently extrapolated to individuals with such variability. It becomes impossible to fit the cephalometric measurements of different individuals into pre-established standard values.^2^ Therefore, the individualization of known means, considering the peculiarities of each ethnic group, becomes quite interesting.

According to the craniofacial growth theory of the Functional Matrix,^3^ the growth of the soft tissues of the face and the execution of breathing and phonation functions, with the passage of air through the pharyngeal spaces, and swallowing would influence the development of the structures of the maxillofacial complex. Therefore, it is reasonable to speculate that patients with different facial features also have different activities of functional matrices and anatomical differences in correlated structures. Regarding the upper airway, the reduction of nasopharyngeal air space can result in mouth breathing. Consequently, the development of dental and dentoskeletal malocclusions, such as maxillary atresia, posterior crossbite, and anterior open bite, may impair function and aesthetics.^4^ ^-^ ^9^ The upper airway space is associated with the growth process of the craniofacial structures and may be related to the etiology of certain malocclusions. However, the literature still lacks information on the variability of the upper airways among different ethnic groups.^10^ ^-^ ^19^

It is known that 3D images have been gaining ground in scientific research; but with some adversities such as high cost and high levels of radiation. Cephalograms, despite being 2D images, are present in the usual orthodontic documentation, facilitating its use in the clinical practice.

Therefore, this study aimed to comparatively evaluate the dimensions of the oropharynx and nasopharynx in Black Caucasian, Caucasian, Black, Japanese Brazilian, and Japanese individuals. The null hypothesis was that the dimensions of the nasopharynx and oropharynx would be similar between the different ethnic groups evaluated.

## MATERIAL AND METHODS

This observational, descriptive, cross-sectional, and retrospective study was approved by the Research Ethics Committee of Bauru School of Dentistry, University of São Paulo (protocol no. 18008819.2.0000.5417). 

The sample size calculation was based on an alpha significance level of 5% and a beta of 20%, to achieve 80% of test power to detect a minimum difference of 1.5mm for the nasopharynx measurement, with a standard deviation of 2.04.^20^ The sample size calculation showed the need for 30 subjects in each group.

The sample was obtained from the Department of Orthodontics files at Bauru School of Dentistry (FOB - USP). An old sample from the Growth Center of this institution was used, in which ethical issues in research were precarious. As it was a convenience sample, no sample calculation was performed. The subjects were selected through photographs of orthodontic documentation and questionnaires administered to guardians that provided information about ancestry (Japanese, European, or African). The following inclusion criteria were adopted: young subjects of both sexes with ages between 11-15 years old; the presence of all permanent teeth up to first molars erupted; and molar Angle Class I relationship. Individuals with crowding greater than 2 mm, with previous orthodontic treatment and/or palatine tonsil and adenoid surgery were excluded.

The total sample comprised 216 subjects, divided into five groups according to race: the Black Caucasian (BC) group comprised 40 descendants of the union of Black and Caucasian (20 female and 20 male), with a mean age of 12.6 years (SD 0.92); Caucasian (C) consisted of 58 subjects (30 female and 28 male), descendants of Caucasian Brazilian of Mediterranean descents (Spanish, Italian, and Portuguese), with a mean age of 13.1 years (SD 0.73); Black (B) consisted of 57 descendants of negroid group origin from the African coast (28 female and 29 males), with a mean age of 12.8 years (SD 0.90); Japanese-Brazilian (JB) comprised 30 descendants of Caucasian parents and Japanese parents and/or grandparents, with a mean age of 13.0 years (SD 1.16); and Japanese (J) consisted of 31 Japanese descendants, except those from the island of Okinawa (18 females and 13 males), with a mean age of 13.1 years (SD 0.56).

**Figure 1:**
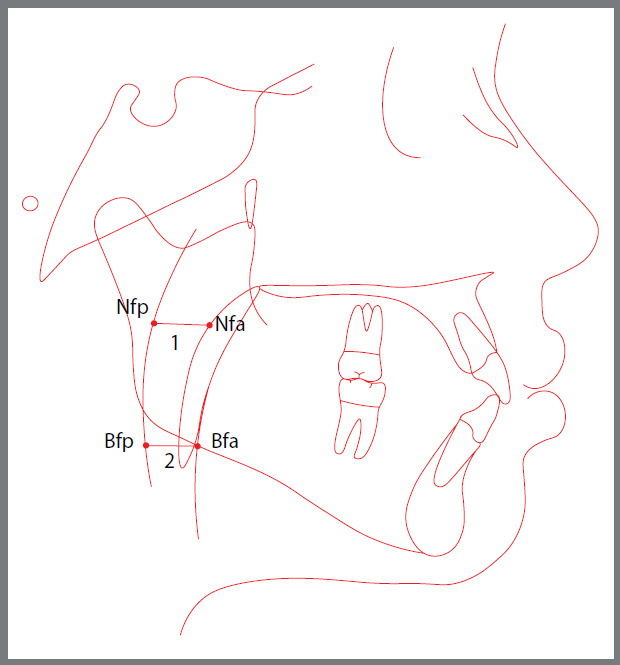
Cephalogram with demarcations of the points relative to the anterior and posterior limits of the nasopharynx (Nfa and Nfp) and oropharynx (Bfa and Bfp), and the respective measured dimensions (1 and 2).

Cephalometric measurements of the upper and lower airways were obtained based on McNamara Jr.’s analysis^21^. The measurements were performed by a single investigator (GZ) using lateral radiographs. The teleradiographs were digitized in JPEG format, using a Scan Maker ui800 scanner (Microtek, Hsinchu, TW), with a resolution of 300 dpi. The Dolphin Imaging program (version 11.9, Dolphin Imaging & Management Solutions, CA, USA) was used for cephalometric tracings. The following cephalometric landmarks were used (Fig 1):

» Anterior nasopharynx (Nfa): the midpoint of the posterior wall of the soft palate).
» Posterior nasopharynx (Nfp): a point on the posterior pharyngeal wall.
» Anterior oropharynx (Bfa): a point of intersection of the posterior border of the tongue with the inferior border of the mandible.
» Posterior oropharynx (Bfp): a point on the posterior pharyngeal wall.

### STUDY ERROR

Thirty radiographs were randomly selected and retraced by the same examiner (GZ) after 30 days of the first measurement. Random errors were calculated according to Dahlberg’s formula (Se² = ∑d²/2n), in which Se² is the error variance, and “d” is the difference between two determinations of the same variable. The systematic error was evaluated with the dependent t-test, with a significance level of *p* < 0,05.

### STATISTICAL ANALYSIS

The Kolmogorov-Smirnov test demonstrated normal distribution for all variables. Analysis of variance (ANOVA) was used for the intergroup comparison, followed by Tukey’s *post-hoc* test. Statistics tests were performed using the Jamovi software (version 1.6, Jamovi stats, Sydney, AU). A statistical significance of 5% was adopted for all tests.

## RESULTS

Random errors were 1.93 and 0.93 for the oropharynx and nasopharynx, respectively. There were no systematic errors (Table 1).

**Table 1:** Random error (Dahlberg formula) and systematic error (t test).

	First Tracing	Second Tracing	Dahlberg	p
	Mean (SD)	Mean (SD)		
Nf	11.13 (3.33)	11.03 (3.37)	0.93	0.67
Bf	12.89 (2.94)	12.32 (2.73)	1.93	0.26

*Statistically significant for p < 0.05. Different letters indicate statistically significant differences.

Nf = Nasopharynx. Bf = Oropharynx.

Caucasian individuals were significantly older than Black Caucasians (Table 2).

**Table 2:** Intergroup comparisons regarding age and sex (Analysis of Variance, followed by Tukey’s test, when applicable).

Age	Black Caucasian (n = 40)	Caucasian (n = 58)	Black (n = 57)	Japanese Brazilian (n = 30)	Japanese (n = 31)	P
	Mean (SD)	Mean (SD)	Mean (SD)	Mean (SD)	Mean (SD)	
	12.6 (0.92)^A^	13.16 (0.73)^B^	12.8 (0.90)^AB^	13.04 (1.16)^AB^	13.16 (0.56)^AB^	0.005*
Sex						
Female	20	30	28	19	18	0.712
Male	20	28	29	11	13	

*Statistically significant for *p* < 0.05.

Different superscript letters indicate statistically significant differences.

The dimension of the nasopharynx was significantly larger in Caucasians, compared to Black Caucasians and Black individuals. The dimension of the oropharynx was similar between the groups (Table 3).

**Table 3:** Intergroup comparisons regarding airway dimensions (Analysis of Variance, followed by Tukey’s test, when applicable).

	Black Caucasian (n = 40)	Caucasian (n = 58)	Black (n = 57)	Japanese Brazilian (n = 30)	Japanese (n = 31)	P
	Mean (SD)	Mean (SD)	Mean (SD)	Mean (SD)	Mean (SD)	
Nf	9.85^A^ (2.04)	12.4^B^ (3.18)	10.6^A^ (2.41)	11.5^AB^ (2.42)	11.2^AB^ (3.02)	< 0.001*
Bf	10.0 (2.75)	11.7 (3.64)	11.1 (4.29)	11.6 (2.97)	11.3 (3.35)	0.093

*Statistically significant for *p* < 0.05.

Different superscript letters indicate statistically significant differences.

Nf = Nasopharynx. Bf = Oropharynx.

## DISCUSSION

The McNamara Jr. analysis evaluates the upper and lower airways, intending to identify a possible obstruction in the airway. Since cephalograms show a 2D image of a 3D structure, it cannot be used for accurate measurements. Furthermore, if the patient is swallowing at the time of image acquisition, the soft palate takes on the appearance of an inverted V, limiting the measurement of the nasopharynx. However, it serves as a warning for possible referral to an otorhinolaryngologist.^21^

According to McNamara Jr.’s analysis,^21^ the average upper airway measurement for adults of both sexes is 17.4 mm, and tends to increase with age. For the lower airways, the average value is 10 to 12 mm, and does not change significantly with age.

No studies were found in the literature comparing the upper airway dimensions of individuals from different races with normal occlusion. It can be suggested that the differences found between the nasopharynx of Caucasians, Black Caucasians, and Blacks may be due to the cranial anthropometric differences of Caucasians and Afro-descendants. Enlow et al.^22^ stated that in Class I cases, the craniofacial patterns are different in Black and Caucasian individuals. In Black individuals, the mandible develops downwards to a greater extent than in Caucasians. Freitas et al.^23^ also concluded that Black Brazilians with normal occlusion have a more protruded maxilla and mandible, greater maxillomandibular discrepancy, and a more horizontal craniofacial growth pattern, when compared to White Brazilian individuals with normal occlusion. The authors also found that Black and Black Caucasians have lower upper posterior facial height, when compared to white individuals, all with normal occlusion.^23^ In addition to cephalometric differences, the length and width of the dental arch of American Blacks are about 10% greater than those of White Americans, and the arch format of Black subjects is more squared and less tapered in the region of premolars and canines.^24^

The nasopharynx linear dimension was significantly larger in Caucasians than in Black Caucasians and Black individuals. Corroborating our results, a previous study measured the oropharyngeal cavities of different races using acoustic pharyngometry, and found that Black Caucasians have the narrowest upper airway.^25^ Perry et al.^26^ related the races and anatomy of the velopharyngeal region through magnetic resonance imaging of Japanese, Caucasians, and Blacks. In this study, Black men obtained the longest and thickest velar measurements, which may be a reason for the reduction of the nasopharynx in black individuals.^26^

Regarding age, Vilella^27^ and Tweed ^28^ stated that the nasopharynx has a similar growth pattern to the rest of the body, and that the nasopharyngeal space increases from 4 to 16 years old. The Black Caucasian sample of the present study had younger patients (12.6) than the Caucasian group (13.16), which could interfere with the average, since the patients in the sample are still growing.^27^ ^,^ ^28^

As limitations of this study, it can be highlighted the airway measurements using two-dimensional cephalograms, and the non-homogeneity of the sample in terms of age. As this was a very old sample, it was not possible to select all patients at the same age. Further studies should use three-dimensional measurements of the upper airways and samples with more homogeneous ages and outside the growth period, for better comparison between groups.

## CONCLUSIONS

The null hypothesis was rejected:

» Caucasian individuals had a significantly larger linear dimension of the nasopharynx than Black Caucasians and Black individuals.
» The linear dimension of the oropharynx was similar among the different ethnic groups.
